# Nomograms for predicting the prognosis of patients with penoscrotal extramammary Paget’s disease: A retrospective study in the SEER database and two medical centers

**DOI:** 10.3389/fonc.2022.973579

**Published:** 2022-10-27

**Authors:** Le-Bin Song, Xiang Zhou, Jiao-Chen Luan, Hao-Yang Wang, Xue-Chen Cao, Jia-Wei Lu, Yu-Jie Zheng, Xin-Feng Wu, Yan Lu

**Affiliations:** ^1^ Department of Dermatology, The First Affiliated Hospital of Nanjing Medical University, Nanjing, China; ^2^ Department of Urology, The First Affiliated Hospital of Nanjing Medical University, Nanjing, China; ^3^ Institute of Dermatology, Chinese Academy of Medical Sciences and Peking Union Medical College, Nanjing, China

**Keywords:** survival, nomogram, SEER, external validation, penoscrotal extramammary Paget’s disease

## Abstract

**Background:**

Extramammary Paget’ s disease (EMPD) is a rare cutaneous malignant tumor, and the prognostic factors associated with penoscrotal EMPD remains unclear. The purpose of this study is to investigate prognostic factors and construct nomograms to predict the outcome of patients with EMPD located in the penis or scrotum.

**Methods:**

From the Surveillance, Epidemiology and End Results (SEER) database, we extracted 95 patients with primary EMPD located in the penis or scrotum as the training cohort. Forty-nine penoscrotal EMPD patients were included from two medical centers as the external validation cohort. Univariate and multivariate Cox regression model were applied to investigating risk factors of cancer-specific survival (CSS) and overall survival (OS). Based on the results of multivariate Cox regression analysis, the nomograms were constructed for predicting CSS and OS of patients with penoscrotal EMPD. The concordance index (C-index), receiver operating characteristic (ROC) curves and calibration curves were applied to evaluate the practicability and accuracy of the nomograms.

**Results:**

In the training cohort, multivariate Cox regression analysis showed that marital status and tumor stage were independent factors of CSS, and marital status, tumor stage and surgery are associated with OS independently in patients with penoscrotal EMPD. Based on these results, we developed nomograms to predict CSS and OS respectively. The C-index values were 0.778 for CSS, and 0.668 for OS in the training set, which displayed the good discriminations. In the external validation set, the C-index values were 0.945 for CSS, and 0.703 for OS. The areas under the curve (AUC) values of nomogram predicting 1-, 3-, and 5-year CSS were 0.815, 0.833, and 0.861 respectively, and 0.839, 0.654, and 0.667 for nomogram predicting 1-, 3-, and 5-year OS respectively. In the validation set, the AUC values of nomogram predicting 1-, 3-, and 5-year CSS were 0.944, 0.896, and 0.896 respectively, and 0.777, 0.762 and 0.692 for nomogram predicting 1-, 3-, and 5-year OS respectively. Additionally, the internal calibration curves also proved that our nomograms have good accuracy.

**Conclusions:**

By incorporating marital status, tumor stage and/or surgery, our nomograms can efficiently predict CSS and OS of patients with penoscrotal EMPD.

## Introduction

Extramammary Paget’s disease (EMPD) is a rare cutaneous malignant tumor, which primarily originate in areas containing abundant apocrine sweat glands. Paget’s disease was firstly identified by James Paget, who described the pathological findings in the nipple lesion of a patient with breast cancer in 1874. Since then, Crocker reported a case of Paget’s disease that affecting the scrotum and penis in 1889 (1). It is estimated that the annual incidence of EMPD is 0.1-2.4 per million (2). EMPD has a high incidence in Asia, reaching 10 per million people, and the incidence rate in men is significantly higher than that in women (3). EMPD patients often present with eczema-like skin lesions in the apocrine area, such as the vulva, penis, scrotum, and perianal areas (4). According to the site of tumor origin, EMPD can be divided into primary neoplasm and secondary neoplasm (5). The secondary EMPD is mostly caused by deep tumor metastasis, including the malignant tumors of the uterus, vagina, colorectal, and urinary system (6). EMPD patients accompanying with other malignant tumors generally have a worse outcome, and the mortality rate is more than 50% (7). Current treatments for EMPD include surgery, topical treatment, radiotherapy, chemotherapy, photodynamic therapy, and targeted therapy. Conventional surgical methods cover local resection, local enlarged resection, Mohs microsurgery, and various modified surgical procedures (8). However, due to lack of early pathological diagnosis, early intervention and standardized treatment, this disease has a high recurrence rate after treatment. Relapsed patients often undertake higher risk of lymph node metastasis and distant organ metastasis, which leads to poor prognosis (9). Meanwhile, EMPD is prone to be misdiagnosed as benign diseases at an early stage, such as eczema and contact dermatitis, which undoubtedly delay definitive diagnosis. It is reported that EMPD has a median delay of 2 years. Penoscrotal EMPD is the most common male EMPD, and patients with penoscrotal EMPD have different clinicopathological features and prognosis compared with other types of EMPD (10). At present, most studies on penoscrotal EMPD are clinical retrospective analysis with small sample sizes. There are no large-sample studies investigating clinicopathological characteristics and prognostic factors of penoscrotal EMPD.

Nomogram has been widely used to predict outcomes of cancer cases by integrating important clinical and pathological characteristics of tumor (11–14). By creating a direct evaluation system, nomogram can assist patients and physicians in making optimal decisions about treatments and prognosis. Furthermore, nomograms have been shown to have a better predictive ability than the traditional TNM classification in several types of cancers (15). However, there is no nomogram to predict prognosis of penoscrotal EMPD. This study aims to develop nomograms to predict the outcome of patients with penoscrotal EMPD based on clinicopathological variables included in Surveillance, Epidemiology and End Results (SEER) database. Moreover, we enrolled penile or scrotal EMPD cases from two medical centers (the First Affiliated Hospital of Nanjing Medical University and the Chinese Academy of Medical Sciences and Peking Union Medical College) as the external validation.

## Materials and methods

### Case data extraction and collection

**Figure 1:**
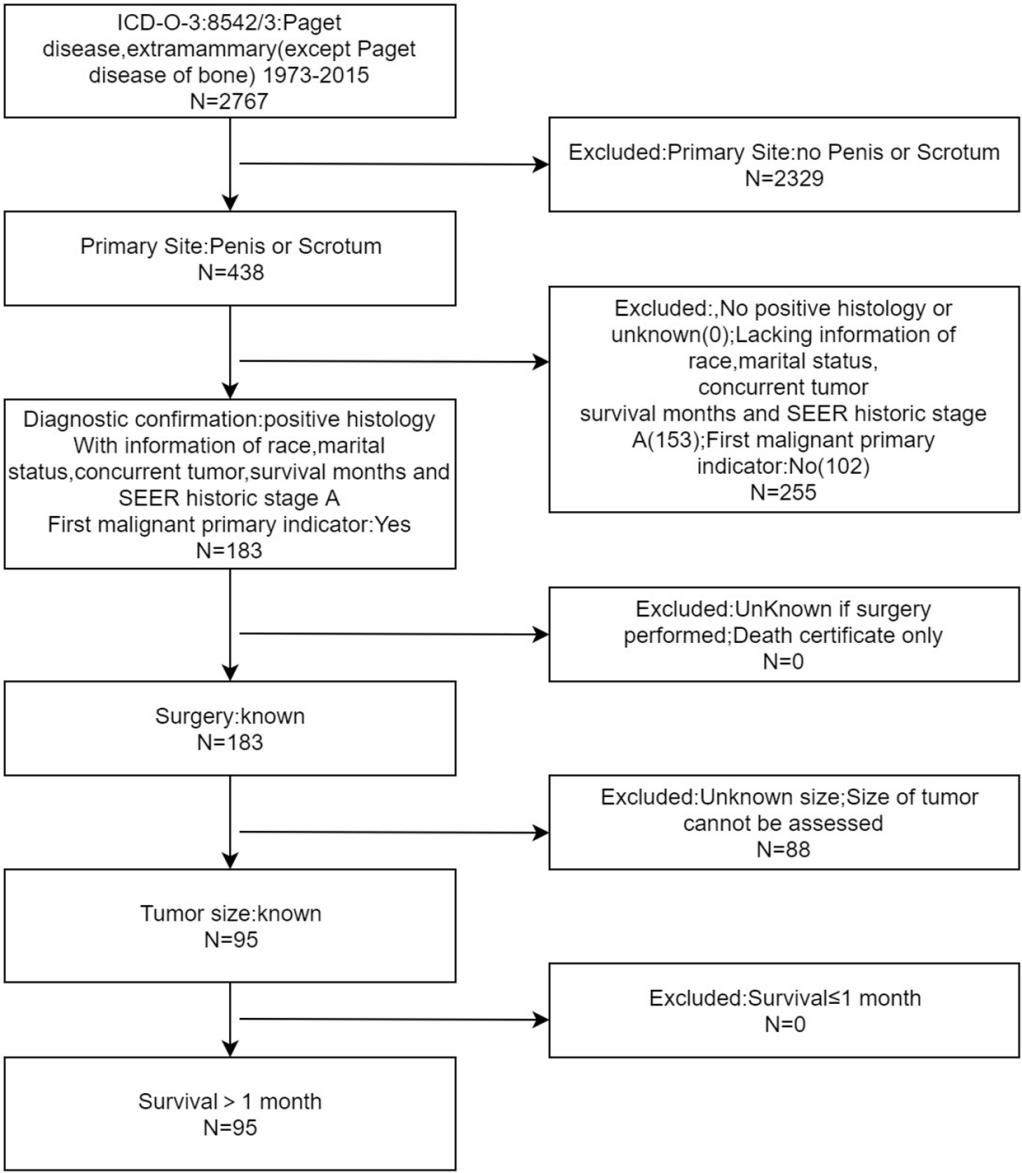
Flow diagram of patients with penoscrotal EMPD enrollment from SEER database.

Firstly, we obtained the clinical data of EMPD patients from the SEER public database, and the screening item was “disease: Paget disease, extramammary (except Paget disease of bone)”. The cases information included patient ID, age at diagnosis, race, marital status, concurrent tumor, primary site, SEER historic stage A, surgery, chemotherapy, survival months, SEER cause-specific death classification and vital status recode. Case inclusion criteria include: (1) EMPD was located in the region of penis or scrotum. (2) EMPD was confirmed by histopathological examination. (3) EMPD was the first-onset tumor in cases. (4) Characteristics of the case, including the year of diagnosis, race, marital status, tumor number, tumor location, SEER historic stage A, surgery information, chemotherapy information, survival month, tumor-specific death status and survival status were available. (5) Survival time of cases were longer than 1 month. The exclusion criteria included: (1) EMPD was not located in the region of penis or scrotum. (2) EMPD was not confirmed by histopathological examination. (3) EMPD was not the first-onset tumor in cases. (4) Patient ID, age at diagnosis, race, marital status, concurrent tumor, primary site, SEER historic stage A, surgery, chemotherapy, survival months, SEER cause-specific death classification, survival status recode was absent. (5) The survival time of cases was less than or equal to 1 month. The case screening flowchart was shown in **Figure 1**. Regarding SEER historic stage A in the SEER database, “local” is defined as an invasive neoplasm confined entirely to the organ of origin. It may include intraluminal extension where specified. “Regional” is defined as a neoplasm that has extended 1) beyond the limits of the organ of origin directly into surrounding organs or tissues; 2) into regional lymph nodes by way of the lymphatic system; or 3) by a combination of extension and regional lymph nodes. “Distant” is defined as a neoplasm that has spread to parts of the body remote from the primary tumor either by direct extension or by discontinuous metastasis. The included cases were utilized as the training cohort to construct the nomogram.

This study also retrospectively analyzed 79 EMPD patients treated in the First Affiliated Hospital of Nanjing Medical University and the Chinese Academy of Medical Sciences and Peking Union Medical College from June 2006 to June 2020. The following patients’ information were included: age, marital status, concurrent tumor, primary site, tumor stage, surgery, chemotherapy, outcome and survival months. All patients received telephone follow-up. Case inclusion and exclusion criteria were consistent with that of EMPD patients from the SEER public database. Finally, a total of 49 cases were included for the present study. Surgical treatment methods include local resection, enlarged local resection, and Mohs microsurgery. The tumor stage of this cohort was converted to SEER historic stage A for further study, based on aforementioned definitions. These cases were used as an external validation cohort. This study was approved by the Ethics Committee of the First Affiliated Hospital of Nanjing Medical University and the Chinese Academy of Medical Sciences and Peking Union Medical College.

### Construction and validation of the nomogram

Nomograms were developed *via* Cox proportional hazards regression models for multivariable time-to-event analysis. Using fitted Cox regression coefficients and variance estimates, weighted estimators are derived for each covariate. In each variable, the highest β coefficient (absolute value) was converted to a scale of 0-100. The points of each variable were then added up to derive total points, which were finally converted to predict CSS and OS in patients with penoscrotal EMPD at 1, 3, and 5 years. The receiver operating characteristic (ROC) curve, the concordance index (C-index) and the calibration curve were utilized to evaluate the prediction accuracy of the nomogram.

### Statistical analysis

Overall survival (OS) is defined as the time from the date of diagnosis to the date of death or the date of the last follow-up. Cancer-specific survival (CSS) is defined as the date from the date of diagnosis to the date of death due to EMPD or the time of the last follow-up date. The univariate and multivariate analysis of the Cox proportional hazard regression models were used to evaluate the correlations between multiple clinicopathological factors and OS along with CSS in EMPD, and calculate the hazard ratio (HR) and 95% confidence interval (CI).

The clinical characteristics and prognostic data of cases in the training group were extracted from the SEER database through the SEER*Stat program (v 8.3.5). SPSS 26.0 (IBM, Armonk, New York, USA) software is used for data statistical analysis, and R3.6.0 (The R Foundation for Statistical Computing, Vienna, Austria) software is used for graphic production. ROC curve was used to investigate the cutoff value of tumor diameter. Continuous variables in clinical characteristics are expressed as mean ± standard deviation. Kaplan–Meier curve, log-rank-test and Cox proportional hazards model were used for survival analysis. P <0.05 was considered statistically significant.

## Results

### Clinicopathological characteristics of patients with penoscrotal EMPD

A total of 95 penoscrotal EMPD patients were obtained from the SEER database, which were applied as the training cohort. In addition, a total of 49 penoscrotal EMPD patients were retrospectively analyzed from the First Affiliated Hospital of Nanjing Medical University and the Dermatology Hospital of the Chinese Academy of Medical Sciences, which were utilized as an external validation set. In the training cohort, 77.89% (74) of the patients were 65 years and older. Whites accounted for 49.47% (47), and Asians and Pacific residents accounted for 50.53% (48). With regard to marital status, 71.58% (68) of the patients were married, and never married cases were 9.47% (9). There were 87.37% (83) of EMPD involved in scrotum, and 12.63% (12) for penis. Most skin lesions (66.32%) were longer than 29.5mm in diameter. For SEER historic stage A, 78.95% (75) of the patients were local stage, 16.84% (16) for regional stage and 4.21% (4) for distant stage. Besides, 86 patients (90.53%) received surgical treatment, and 8 patients (8.42%) were treated with chemotherapy. In the external validation set, 81.63% (40) of patients were 65 years or older, and 69.39% (34) of patients were married. There were 41 (83.67%) case undertaking scrotal EMPD and 16.33% (8) of cases suffering penile lesion. More than half (67.35%) of tumor diameters were longer than 29.5 mm. For SEER historic stage A, 73.47% (36) of the patients were local stage, 20.41% (10) for regional stage and 6.12% (3) for distant stage. A total of 45 patients (91.84%) received surgery, and 4 patients (8.16%) received chemotherapy. The population information, tumor characteristics and treatment details in the training and the external validation cohort were shown in **Table 1**.

**Table 1:** The clinical characteristics of the training cohort and the external validation cohort.

Clinical characteristics	Training cohort (n = 95), n (%)	External validation cohort (n = 49), n (%)
**Age at diagnosis**		
<65	21 (22.11%)	9 (18.37%)
65-74	38 (40.00%)	21 (42.86%)
≥75	36 (37.89%)	19 (38.77%)
**Race**		
White	47 (49.47%)	0 (0%)
Asian or Pacific Islander	48 (50.53%)	49 (100%)
**Marital status**		
Married	68 (71.58%)	34 (69.39%)
Never married	9 (9.47%)	7 (14.28%)
Other^*^	18 (18.95%)	8 (16.33%)
**Concurrent tumor**		
≤1	75 (78.95%)	39 (79.59%)
>1	20 (21.05%)	10 (20.41%)
**Primary site**		
Scrotum	83 (87.37%)	41 (83.67%)
Penis	12 (12.63%)	8 (16.33%)
**Tumor size**		
≤29.5mm	32 (33.68%)	16 (32.65%)
>29.5mm	63 (66.32%)	33 (67.35%)
**SEER historic stage A**		
Localized	75 (78.95%)	36 (73.47%)
Regional	16 (16.84%)	10 (20.41%)
Distant	4 (4.21%)	3 (6.12%)
**Surgery**		
No	9 (9.47%)	4 (8.16%)
Yes	86 (90.53%)	45 (91.84%)
**Chemotherapy**		
No	87 (91.58%)	45 (91.84%)
Yes	8 (8.42%)	4 (8.16%)
**Survival time, month (range)**	90.0 (5- 352)	49.9 (2-126)

^*^Other: divorced, separated, or widowed.

SEER, Surveillance, Epidemiology and End Results.

### Prognostic factors related to OS and CSS in patients with penoscrotal EMPD

**Figure 2:**
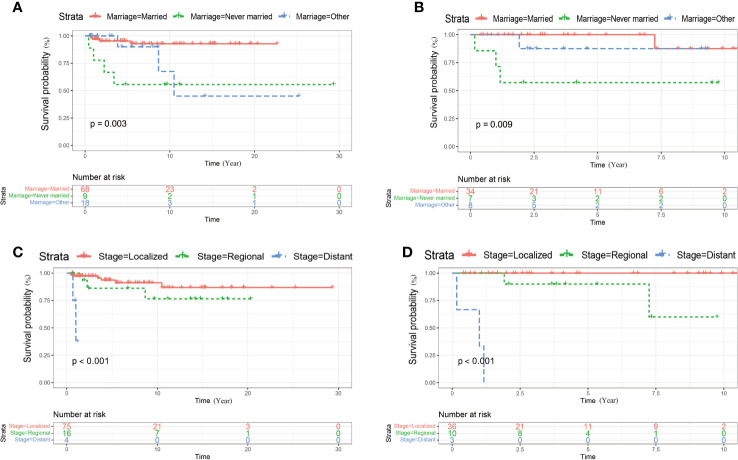
Kaplan–Meier curves of CSS for patients with penoscrotal EMPD stratified by marital status **(A, B)** and SEER historic stage A **(C, D)** in thetraining cohort and validation cohort respectively.

**Figure 3:**
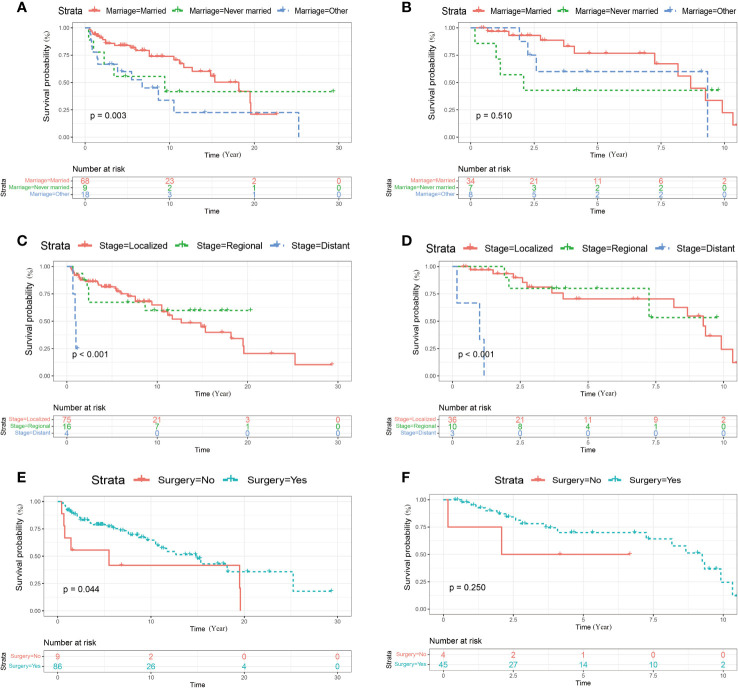
Kaplan–Meier curves of OS for patients with penoscrotal EMPD stratified by marital status **(A, B)**, SEER historic stage A **(C, D)** and surgery **(E, F)** in the training cohort and validation cohort respectively.

Kaplan–Meier curve and log-rank-test demonstrated that never married status and high tumor stage were related to worse CSS of cases in the training and the external cohort (**Figures 2A–D**). The associations between marital status, tumor stage along with surgery and OS were also explored by Kaplan–Meier curve and log-rank-test in the training and the external cohort (**Figures 3A–F**). In the training cohort, the marital status (*P* = 0.003), SEER historic stage A (*P* = 0.000), and surgery (*P* = 0.044) were significantly associated with OS (**Figures 3A, C, E**). In the validation set, SEER historic stage A (*P* = 0.000) was related to OS with significance (**Figure 3D**). However, the relationships between marital status (*P* = 0.510) along with surgery (*P* = 0.250) and OS were not statistically significant (**Figures 3B, F**).

In the training cohort, multivariate Cox regression analysis showed that marital status (never married vs married: HR 5.646; 95% CI, 1.287- 24.779) and stage (distant vs local: HR 23.893; 95% CI, 2.791-204.552) were independent predictors of CSS in patients with penoscrotal EMPD. However, age, race, concurrent tumor, primary tumor site, tumor size, surgery, and chemotherapy are not independent factors of CSS (**Table 2**). Meanwhile, multivariate Cox regression analysis showed that marital status (other: divorced, separated, or widowed vs married: HR 3.091; 95%CI, 1.479-6.461), stage (distant vs local: HR 11.836; 95%CI, 2.852-49.125) and surgery (yes vs no: HR 0.358; 95%CI, 0.148-0.864) are independent predictors of OS in patients with penoscrotal EMPD. However, we found that age, race, concurrent tumor, primary tumor site, tumor size, and chemotherapy are not independent factors of OS (**Table 3**).

**Table 2:** Univariate and multivariate analyses of CSS in the training cohort.

Clinical characteristics	No. of patients	Univariate analysis			Multivariate analysis		
		HR	95%Cl	p-value	HR	95%Cl	p-value
**Age at diagnosis**							
<65	21	1.0					
65-74	38	0.492	0.099-2.451	0.387			
>74	36	1.513	0.355-6.453	0.576			
**Race**							
White	47	1.0					
Asian or Pacific Islanderr	48	1.796	0.523-6.161	0.352			
**Marital status**							
Married	68	1.0			1.0		
Never married	9	8.258	2.056-33.121	0.003	5.646	1.287-24.779	0.022
Other^*^	18	3.566	0.795-16.001	0.097	4.130	0.903-18.898	0.068
**Concurrent tumor**							
≤1	75	1.0					
>1	20	0.244	0.031-1.943	0.183			
**Primary site**							
Scrotum	83	1.0					
Penis	12	0.039	0.000-59.212	0.386			
**Tumor size**							
≤29.5mm	32	1.0					
>29.5mm	63	5.514	0.705-43.107	0.104			
**SEER historic stage A**							
Localized	53	1.0			1.0		
Regional	38	2.037	0.506-8.209	0.317	1.452	0.344-6.124	0.612
Distant	4	32.962	4.366-248.844	0.001	23.893	2.791-204.552	0.004
**Surgery**							
No	9	1.0					
Yes	86	0.337	0.073-1.567	0.165			
**Chemotherapy**							
No	87	1.0					
Yes	8	1.125	0.144-8.810	0.911			

^*^Other: divorced, separated, or widowed.

**Table 3:** Univariate and multivariate analyses of OS in the training cohort.

Clinical characteristics	No. of patients	Univariate analysis			Multivariate analysis		
		HR	95%Cl	p-value	HR	95%Cl	p-value
**Age at diagnosis**							
<65	21	1.0					
65-74	38	1.031	0.423-2.514	0.947			
>74	36	2.367	0.953-5.879	0.063			
**Race**							
White	47	1.0					
Asian or Pacific Islanderr	48	1.913	0.995-3.679	0.052			
**Marital status**							
Married	68	1.0			1.0		
Never married	9	1.640	0.612-4.396	0.325	1.501	0.536-4.206	0.440
Other^*^	18	2.504	1.229-5.102	0.012	3.091	1.479-6.461	0.003
**Concurrent tumor**							
≤1	75	1.0			1.0		
>1	20	0.561	0.261-1.206	0.139	0.436	0.190-1.001	0.050
**Primary site**							
Scrotum	83	1.0					
Penis	12	0.451	0.139-1.470	0.187			
**Tumor size**							
≤29.5mm	32	1.0					
>29.5mm	63	1.191	0.601-2.358	0.617			
**SEER historic stage A**							
Localized	53	1.0			1.0		
Regional	38	0.781	0.324-1.882	0.581	0.982	0.392-2.457	0.968
Distant	4	10.720	2.742-41.908	0.001	11.836	2.852-49.125	0.001
**Surgery**							
No	9	1.0			1.0		
Yes	86	0.433	0.187-1.002	0.051	0.358	0.148-0.864	0.022
**Chemotherapy**							
No	87	1.0					
Yes	8	1.461	0.567-3.768	0.433			

^*^Other: divorced, separated, or widowed.

OS, overall survival; HR, hazard ratio.

### Construction and verification of the nomograms

**Figure 4:**
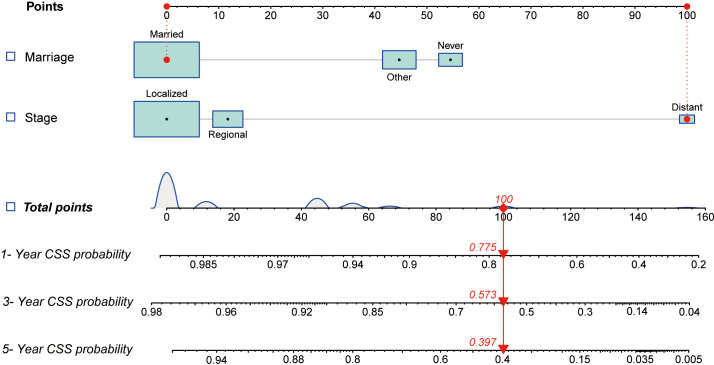
The nomogram for predicting 1-year, 3-year, and 5-year CSS of patients with penoscrotal EMPD.

**Figure 5:**
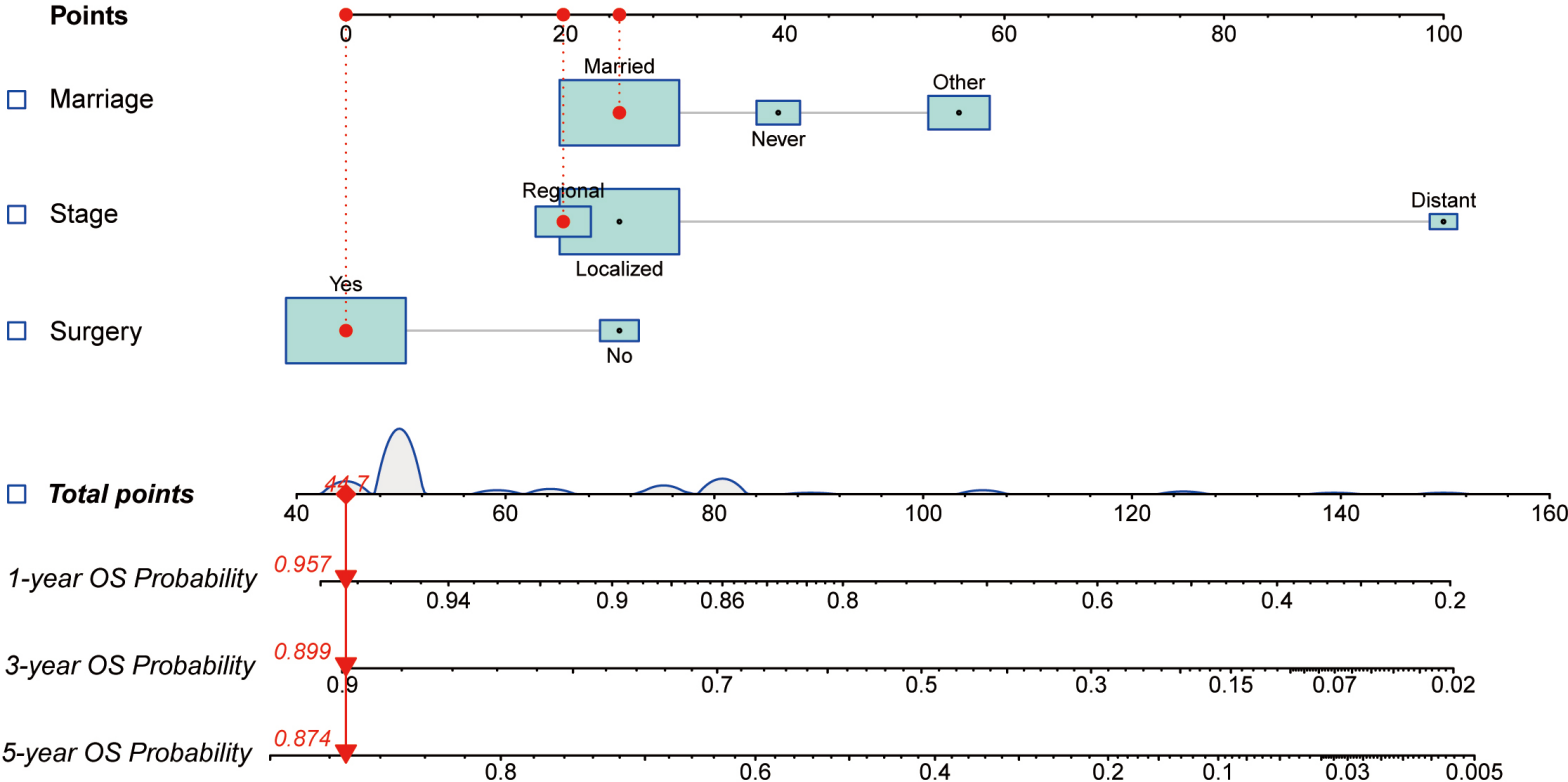
The nomogram for predicting 1-year, 3-year, and 5-year OS of patients with penoscrotal EMPD.

All independent factors demonstrated by the Cox regression analysis were utilized to construct nomograms for predicting the survival of patients with penoscrotal EMPD. In our study, marital status and SEER historic stage A were included in the nomogram to predict 1-year, 3-year, and 5-year CSS, and marital status, surgery, and SEER historic stage A were utilized to construct the nomogram to predict 1-year, 3-year, and 5-year OS (**Figures 4**, **5**). The nomogram was used by adding the score of each point of the variables to get the total score, which corresponded to predicted 1-year, 3-year, and 5-year CSS or OS rate.

**Figure 6:**
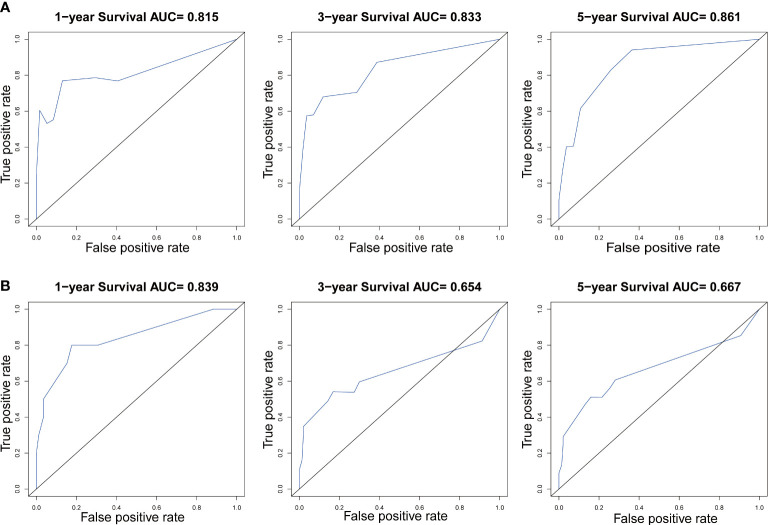
The ROC curves of the nomogram predicting 1-year, 3-year, and 5-year **(A)** CSS and **(B)** OS in the training cohort.

**Figure 7:**
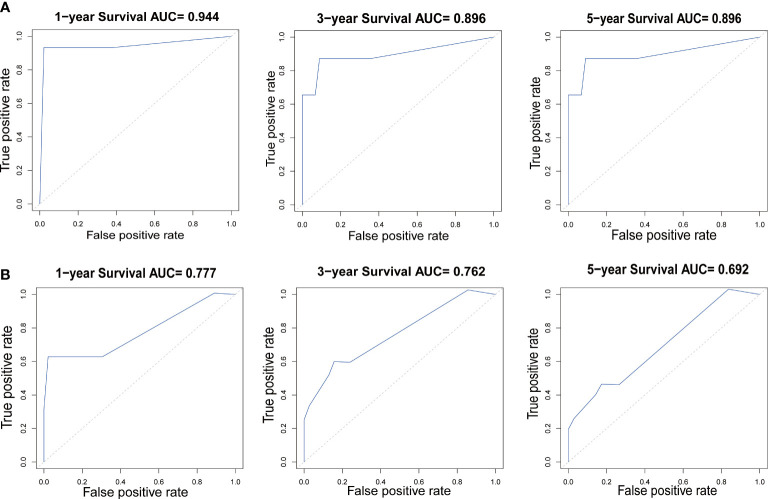
The ROC curves of the nomogram predicting 1-year, 3-year, and 5-year **(A)** CSS and **(B)** OS in the validation cohort.

**Figure 8:**
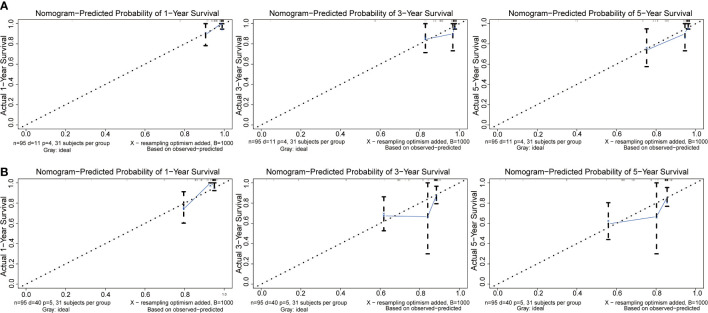
The calibration curves of the nomogram predicting 1-year, 3-year, and 5-year **(A)** CSS and **(B)** OS in the training cohort.

In our study, nomograms were verified internally and externally. The C-index values were 0.778 for CSS and 0.668 for OS in the training set respectively, which displayed the good discriminations. In the validation set, the C-index values were 0.945 for CSS and 0.703 for OS respectively. Additionally, the ROC curves were utilized to assess the efficiency of the nomograms. In the training set, the AUC values of nomogram predicting 1-, 3-, and 5-year CSS were 0.815, 0.833, and 0.861 respectively, and 0.839, 0.654, and 0.667 for nomogram predicting 1-, 3-, and 5-year OS respectively (**Figure 6**). We also plotted the ROC curves in the validation set, which revealed that the AUC values of nomogram predicting 1-, 3-, and 5-year CSS were 0.944, 0.896, and 0.896 respectively, and 0.777, 0.762 and 0.692 for nomogram predicting 1-, 3-, and 5-year OS respectively (**Figure 7**). In addition, the internal calibration curve for CSS and OS also proved that our nomograms have good accuracy (**Figure 8**).

## Discussion

EMPD, a relatively rare skin malignant tumor, are mostly located in areas full of apocrine glands, such as the vulva, perianal, groin, penis and scrotum, accounting for 6.5% of all cutaneous Paget’s disease (16). Recent studies have suggested that penoscrotal EMPD take up for approximately 17.3% of all EMPD and 47.9% of all male EMPD (17). The main clinical manifestations of penoscrotal EMPD are pruritus and pain, and wide local excision is first choice (18). However, due to low incidence and non-typically clinical symptoms, penoscrotal EMPD is frequently incorrectly diagnosed as a benign disease at an early stage (19). Limited studies showed that symptom duration, distant metastasis, depth of invasion, and HER2/neu are related to recurrence after operation in patients with penoscrotal EMPD. For survival, a few studies focused on the survival of patients with penoscrotal EMPD, and the predictive prognosis factors remain unclear (20). Therefore, we conducted this research to clarify potential prognostic factors in penoscrotal EMPD patients and develop the nomogram to predict prognosis of penoscrotal EMPD cases.

A total of 49 penoscrotal EMPD cases from two medical centers were included in this study, as the external validation cohort. There were 81.63% of patients older than 65 years. Ninety-five penoscrotal EMPD cases from SEER database were finally enrolled in the present research, as training cohort, 77.89% of them older than 65 years. Our results revealed that penoscrotal EMPD is more likely to occur in relatively older patients. Additionally, 21.05% and 20.41% of patients had concurrent tumor in the training and external validation cohort respectively. The concurrent tumor in penoscrotal EMPD cases were involved in uterus, vagina, colorectal and urinary system, which suggests that there is a certain associations between penoscrotal EMPD and other tumors. Therefore, when focusing on the treatment of EMPD patients, it is also necessary to pay attention to whether the patient has other tumors in clinical practice.

Nowadays, marital status is one of the necessary factors of social relationships (21, 22). A clinical study involving 300,000 breast cancer patients demonstrated that the OS and CSS of the married group with breast cancer were better than the unmarried group and the divorced/separated/widowed group, which is consistent with our results (23). This phenomenon may be related to the fact that married patients have better financial support, complete medical insurance, and systematic treatment. For example, the proportion of married patients receiving surgery is higher than that of the other two group. In penoscrotal EMPD patients, a good marital status suggests a better prognosis, which is consistent with the conclusions of existing studies.

The depth of tumor invasion and tumor thickness are also recognized as the important factors in the prognosis of EMPD patients (24, 25). EMPD confined to the epidermis generally indicates a better prognosis, while patients undertaking deeper tumor invasion wound have a worse prognosis, due to the risk of tumor metastasis. Several studies demonstrated that there were significant differences in prognosis between case with EMPD limited to the papillary dermis and infiltrating into reticular dermis or deeper tissue (26, 27). There are studies also showing that the prognosis of patients with tumor invasion depth less than 1mm is relatively well, while patients with tumor invasion depth greater than 4mm suffered relatively poor prognosis (28). Lesions confined to the epidermis are considered to have a lower risk of metastasis in EMPD, the deeper the tumor infiltration always indicts a higher risk of distant metastasis, a higher the fatality rate, and a poor prognosis.

Lymph node metastasis is an independent risk factor in EMPD patients (28, 29). Enlarged lymph nodes in physical examination or imaging examinations detecting lymph node metastasis usually imply a poor prognosis (30). Our study showed that pathologically positive lymph node is remarkably related to the patient’s outcome. In the meantime, nodules in the primary tumor, deeper tumor invasion, and invasion of surrounding lymphatics and blood vessels appear in penoscrotal EMPD patients with positive lymph node biopsy. Our study also demonstrated that regional and distant metastasis are crucial factors in prognosis of patient. In the training cohort, 20% of patients with regional and distant metastasis died of EMPD, and 38.5% in the external validation cohort. Our results demonstrated lymph node biopsy is very significant in clinical practice. The sentinel lymph node biopsy plays an important role in the detection of lymph node metastasis in patients with EMPD, which is also can be used in the early stages of EMPD to detect lymph node metastasis (31, 32). Besides, the number of positive lymph nodes has also been shown to be associated with prognosis of patients (33). There is a significant difference in survival between patients with zero or one positive lymph node metastasis and more than two positive lymph nodes (34). Higher levels of serum CEA and CYFRA were predictive factors of lymph node metastasis with high sensitivity in EMPD (35). HER-2 gene enrichment can be applied as a biomarker for lymph node metastasis in patients with EMPD (36). Moreover, ultrasound, ^18^F-fluorodeoxyglucose (FDG) positron emission tomography and computed tomography can be used to detect early lymph node metastasis (37). For penoscrotal EMPD patients, early lymph node examination and surgery may improve the outcome of patients.

The recurrence rate and mortality of EMPD are associated with therapeutic methods (38). Wide local excision is the most commonly performed, accounting for approximately 57% of all therapeutic methods in clinical practice, followed by local imiquimod (26%) and Mohs microsurgery (26%) (39). Furthermore, radiotherapy and chemotherapy can also be applied for the treatment of EMPD, but treatment respond vary obviously (40). Due to the limited sample size in our study, we can not conclude that whether chemotherapy was a predictive factor of OS in penoscrotal EMPD patients including early and advanced setting. At present, surgical treatment is the first choice for the treatment of penoscrotal EMPD, and the mortality rate of patients undergoing surgery has been shown to be significantly lower than that of patients who have not receive surgery (41). Meanwhile, as shown in our study, surgical treatment is demonstrated as an independent predictor of OS, which is consistent with the conclusions of published studies. However, surgical treatment is not an independent predictor of CSS in the present study, which may be due to the limited number of cases. The current surgical treatment methods include local resection, wide local excision, Mohs microsurgery and various modified surgical methods (42). Among of them, Mohs microsurgery is considered to have a lower recurrence rate and risk of metastasis compared with other traditional surgical methods, which is related to rapid pathology applied during Mohs microsurgery and undefined margin of local resection and wide local excision (43). Nowadays, there is still no golden standard for EMPD surgery, postoperative patients still suffer from local recurrence and distant metastasis.

Generally, this is one of the first studies to develop nomograms to predict prognosis of penoscrotal EMPD patients through the SEER database, and verified by external data by cases from two medical centers. However, there is no denying that our research had some limitations. First of all, some clinical information is not included in the SEER database, such as immunohistochemistry and results of imaging examinations, which limits the comprehensive analysis in the prognostic risk factors of EMPD patients. Secondly, the cases included in our studies were limited.Large sample, multi-center studies are needed to verified our results.

## Conclusion

This is one of the first studies investigating the risk factors of prognosis in patients with penoscrotal EMPD, and successfully develop nomograms for predict outcome of patients. By incorporating marital status, tumor stage, and operative treatment status, our nomogram can effectively predict CSS and OS in patients with penoscrotal EMPD.

## Data availability statement

The raw data supporting the conclusions of this article will be made available by the authors, without undue reservation.

## Ethics statement

This study was approved by the Ethics Committee of the First Affiliated Hospital of Nanjing Medical University and the Chinese Academy of Medical Sciences and Peking Union Medical College [2021-SR-244]. The patients/participants provided their written informed consent to participate in this study.

## Author contributions

X-FW, YL: Protocol/project development. L-BS, XZ, J-CL: Data collection or management. L-BS, XZ, J-CL: Data analysis; H-YW, X-CC, Y-JZ, J-WL: Manuscript writing/editing. All authors read and approved the final manuscript. All authors contributed to the article and approved the submitted version.

## Funding

This article was funded by the National Natural Science Foundation of China [grant number: 81872541].

## Acknowledgments

We would like to thank the researchers and study participants for their contributions.

## Conflict of interest

The authors declare that the research was conducted in the absence of any commercial or financial relationships that could be construed as a potential conflict of interest.

## Publisher’s note

All claims expressed in this article are solely those of the authors and do not necessarily represent those of their affiliated organizations, or those of the publisher, the editors and the reviewers. Any product that may be evaluated in this article, or claim that may be made by its manufacturer, is not guaranteed or endorsed by the publisher.
